# Simultaneous Visualization of R-Loops/RNA:DNA Hybrids and Replication Forks in a DNA Combing Assay

**DOI:** 10.3390/genes15091161

**Published:** 2024-09-03

**Authors:** Miroslav Penchev Ivanov, Heather Zecchini, Petra Hamerlik

**Affiliations:** 1 Early Oncology Bioscience, AstraZeneca, Cambridge CB2 0AA, UK; miroslav.ivanov@crick.ac.uk; 2 The Francis Crick Institute, London NW1 1AT, UK; 3 Light Microscopy Facility, University of Cambridge, Cancer Research UK Cambridge Institute, Cambridge CB2 0RE, UK; heather.zecchini@cruk.cam.ac.uk; 4 Division of Cancer Sciences, University of Manchester, Manchester M13 9PL, UK

**Keywords:** R-loops, DNA combing, assay development

## Abstract

R-loops, structures that play a crucial role in various biological processes, are integral to gene expression, the maintenance of genome stability, and the formation of epigenomic signatures. When these R-loops are deregulated, they can contribute to the development of serious health conditions, including cancer and neurodegenerative diseases. The detection of R-loops is a complex process that involves several approaches. These include S9.6 antibody- or RNAse H-based immunoprecipitation, non-denaturing bisulfite footprinting, gel electrophoresis, and electron microscopy. Each of these methods offers unique insights into the nature and behavior of R-loops. In our study, we introduce a novel protocol that has been developed based on a single-molecule DNA combing assay. This innovative approach allows for the direct and simultaneous visualization of RNA:DNA hybrids and replication forks, providing a more comprehensive understanding of these structures. Our findings confirm the transcriptional origin of the hybrids, adding to the body of knowledge about their formation. Furthermore, we demonstrate that these hybrids have an inhibitory effect on the progression of replication forks, highlighting their potential impact on DNA replication and cellular function.

## 1. Introduction

Transcription and replication are fundamental processes that occur on the same DNA template, typically separated both temporally and spatially. However, this dichotomization is not always feasible, particularly for S phase-specific genes that must be expressed concurrently with active replication [1]. Additionally, the transcription of long genes can extend beyond the G1 phase, continuing through the S-phase or even spanning the entire cell cycle. Transcription impedes replication progression through direct collisions, torsional stress, or the formation of RNA:DNA hybrids known as R-loops. These R-loops, formed by the hybridization of nascent RNA with the template DNA, leave single-stranded non-template DNA exposed [1].

The physiological functions of R-loops include the regulation of transcription and class-switch recombination [1,2,3,4]. R-loops have been associated with repeat expansion disorders such as Fragile X, Huntington’s, and myotonic dystrophy [5,6]. Mutations in R-loop processing factors and increased R-loop levels have been identified in diseases, e.g., Aicardi–Goutières syndrome (mutations in TREX1 or RNAse H2) [7], as well as ataxia with oculomotor apraxia type 2 and in myotrophic lateral sclerosis ALS4 (mutations in Senataxin) [3]. Deregulated transcription, epigenetic changes, and splicing perturbations can cause increased R-loops, resulting in elevated replication stress, transcription-replication conflicts, double-stranded DNA breaks, and genome instability [8] which are hallmarks of ageing and various diseases, including cancer and neurodegeneration [4,7,9]. In addition to proteins specialized to unwind R-loops, such as RNAse H1/2 and SETX, multiple tumor suppressor genes have been shown to prevent R-loop accumulation. Such proteins are P53, BRCA1, BRCA2, BLM, WRN, FANCD2, FANCM, SMARCAL1, ZRANB3 [9,10,11,12,13,14,15,16,17]. The abundance of tumor suppressors involved in R-loop regulation demonstrates the profound importance of R-loop level management for genome integrity and cellular fitness.

Given the emerging role of R-loops in human disease, there is a critical need for methods to quantify and visualize them in the context of ongoing DNA replication. Traditional studies have relied on population-level correlations between R-loop levels and markers of replication stress. Recently, electron microscopy (EM) has enabled the direct visualization of R-loops and replication forks at the single-molecule level [18]. However, EM techniques have limitations, including restricted detection distances and the potential for small hybrids to escape detection.

In this study, we present a novel protocol for the direct and simultaneous visualization of RNA:DNA hybrids and replication forks using a DNA combing assay. This method offers several advantages: it is technically accessible to the broader scientific community, differentiates replication phenotypes at single DNA fiber resolution, and provides relatively high throughput. Moreover, it overcomes the inherent limitations of EM, allowing for a more comprehensive analysis of R-loop formation in the context of replication.

## 2. Materials and Methods

The reagents (Table 1) and equipment (Table 2) used in this protocol are listed below.

### 2.1. Step-by-STEP PROTOCOL

Timing from cell labeling to combing staining: 5 days.

#### 2.1.1. Cell Labelling

Timing: 1.5 h

Note: This protocol is based on using adherent cell culture. It can be modified for suspension cells.
Note: The length of nucleotide analogue incorporation can vary depending on the cell type. It is typically 20–30 min each.
Note: Plate cells for experiments 24–48 h before labelling. Cells should be less than 70% confluent and exponentially growing on the day of the experiment.
Note: Before starting, equilibrate the medium in a cell culture incubator (37 °C and 5% CO_2_)

1. Add CldU to the growing cells at a final concentration of 25 µM (25 mM stock in DMSO). Mix gently and place back in an incubator for 25 min.
2. Remove the cell medium and quickly rinse once with fresh pre-equilibrated medium. Remove the rinse medium.
3. Add fresh pre-equilibrated medium containing 100 µM IdU (25 mM stock in DMSO). Incubate for 25 min. [Note: the concentration and order of the nucleotide analogues incubations can vary depending on purpose, cell type, and staining quality.]
4. Remove IdU-containing medium.
5. Add ice-cold PBS to cells.
6. Remove PBS and dissociate cells using Trypsin, TriplE, or another appropriate dissociation method. Plates can be placed back in an incubator for 2–3 min to facilitate detachment from the plastic.
7. Harvest cells in cold PBS and keep cell suspensions on ice. [Note: if handling multiple samples, cells can be left on ice at this stage for up to 20 min until all samples are harvested.]
8. Determine cell concentration using cell counter.

#### 2.1.2. Preparing DNA in an Agarose Plug

Timing: 1 h + overnight

Note: use 25,000–50,000 cells per plug. e.g., if you need 4 plugs of 25,000 cells each, transfer suspension containing 100,000 cells
Note: This protocol closely follows the manual of Genomic Vision FiberPrep^®^ DNA extraction kit.

9. Transfer enough cells for the desired number of plugs to a new 1.5-mL tube and spin them down in a tabletop centrifuge. Remove the supernatant.
10. Resuspend the cell pellet in 40 µL/plug buffer 1 (e.g., for 4 plugs, use 160 µL buffer 1) and leave suspension at room temperature.
11. Add 50 μL of melted buffer 2 (e.g., for 4 plugs, use 200 µL buffer 2; Melt at 68 °C for 10 min and keep it melted at 50 °C).
12. Mix well by pipetting and immediately fill 90 µL/plug into disposable mould.
13. Leave it to solidify at 4 °C for 15 min.
14. Prepare mix of buffer 3 and component 3 in ratio 9:1. Use 250 µL mix per plug. [Note: Use a 2-mL tube for up to 4 plugs. Use 5- or 15-mL tube or more plugs.]
15. Incubate at 50 °C overnight with gentle shaking.
16. Wash the plugs in 15 mL 1× buffer 4: 3 times 1 h with rotation. [Note: spare plugs can be stored in buffer 5 (1 mL/plug) at 4 °C, protected from light for up to 1 year.]

#### 2.1.3. Preparing DNA Solution for Molecular Combing

Timing: 1–3 days

Note: Before starting, set up two thermo blocks or water baths to 68 °C and 42 °C. If the plug is stored in buffer 5, wash it in 15 mL 1X buffer 4 for a couple of hours.

17. Transfer one plug to a 2-mL tube.
18. Add 1 mL buffer 7 and incubate at 68 °C for 20 min. [Note: From this point, DNA is in solution. Handle with extreme care to avoid mechanical shearing!]
19. Carefully transfer the tube at 42 °C and let equilibrate for 5 min
20. Add 1.5 µL of component 7 (β-agarase). Do not mix.
21. Incubate overnight at 42 °C.
22. Add 1200 µL buffer 7 in a disposable reservoir.
23. Gently pour the DNA solution into the reservoir. [Note: We recommend leaving the solution at 4 °C for at least 24 h before combing.]

#### 2.1.4. Molecular Combing

Timing: 1.5–2 h

Use Genomic Vision FiberComb device and combing coverslips (silanized coverslips)

24. Place the DNA reservoir, equilibrated to room temperature, in the combing device.
25. Attach one or 2 coverslips on the holders.
26. Push the button, the slide goes in the solution, and after 5 min it is pulled out of the solution at a constant speed of 300 µm/s.
27. Remove coverslips and place them on a staining rack.
28. Bake in an oven at 60 °C for 40–60 min.
29. Slides can either be processed for immunodetection or stored at −20 °C.

Note: One additional coverslip per condition should be prepared for combing quality check: Immerse these in 100 nM YOYO dye in PBS for 5 min, rinse with water, air-dry, mount the coverslips on microscopy slides and image. Long intact and high density (but not overlapping) DNA strands indicate good quality genomic DNA prep and successful combing.

#### 2.1.5. Immuno-Staining

Timing: 1.5 days

Note: all antibody incubations and the RNAse A treatment are performed by flipping the coverslip over a drop (20–50 µL/coverslip) of solution on a glass slide. We recommend cleaning the glass slides with 70% ethanol to increase surface tension and avoid overspreading of the drops. All washing, blocking, fixation, denaturation, and dehydration steps are performed on a staining rack in a beaker.
Note: ‘Blocking buffer’: 1% BSA + 0.1% Tween-20 in PBS.

30. Wash once in dH2O (staining rack, fast wash).
31. Wash once in PBS (staining rack, fast wash).
32. Immerse in 1% Triton-PBS for 5 min at room temperature (RT).
33. Wash twice in PBS (staining rack, fast wash).
34. Treat with RNAse A for 1 h at 37 °C on glass slides in a humidity chamber: 170 mM NaCl, 0.1 mg/mL RNAse A, blocking buffer [Note: PBS in the blocking buffer contains 137 mM NaCl, adding to a total of about 300 mM NaCl to avoid degrading R-loops by RNAse A [19]].
35. Place coverslips on a staining rack and rinse with PBS.
36. Transfer the rack with coverslips in blocking buffer for 1 h at RT.
37. Primary S9.6 antibody staining: Add 40 μL/slide 1:500 dilution of mouse anti-S9.6 antibody in blocking buffer. Incubate in humidity chamber overnight at 4 °C, coverslip flipped on a microscopy glass slide.

CRITICAL: The success of the R-loop staining depends strongly on the quality of the S9.6 antibody. We recommend testing different S9.6 samples in parallel to identify a working batch by following the protocol down to step 47 and staining dsDNA with YOYO. The appearance of the characteristic clusters of R-loop dots on DNA (Figure S1C) indicates an S9.6 sample that can be used for staining. See also Section 4 for further details and confirmed antibody batches.

38. Leave slides in cold PBS for 5 min.
39. Wash with blocking buffer for 15 min.
40. Wash 2 min in PBS.
41. Fix with 4% Formaldehyde for 10 min RT.
42. Wash with PBS for 2 min.
43. Wash with blocking buffer for 5 min.
44. Secondary antibody staining: Add 50 μL 1:250 anti-mouse AF-647 in blocking buffer. Incubate for 1.5 h at RT.
45. Wash 5 min in PBS.
46. Wash 10 min in blocking buffer at RT, shake gently.
47. Rinse briefly in PBS.

Note: At this point, the quality of S9.6 staining can be tested: immerse the coverslips in 100 nM YOYO dye in PBS for 5 min, rinse with water, air-dry, mount the coverslips on microscopy slides and image them.

48. Fix with 4% Formaldehyde in PBS for 10 min RT.
49. Wash once briefly in PBS.
50. Wash once with blocking buffer for 5 min at RT.
51. Wash for 2 min in PBS.
52. Denature combed DNA with 0.5M NaOH/1M NaCl for 30 min at RT.

Note: As NaOH degrades RNA, covalent cross-linking of the antibodies to the binding regions in steps 41 and 48 creates a replica of the hybrids that can persist after denaturation.

53. Wash in PBS for 5 min.
54. Dehydrate by immersing the racks with coverslips in a sequence of 70–90–100% ethanol, 30–60 s in each, and air-dry.
55. Block slides with blocking buffer, 15–30 min at RT.
56. Incubate with replication fork primary antibodies for 1 h at RT:

Note: Fork staining is performed in BlockAid, 40 μL/coverslip (if necessary, the staining volume can be reduced to 20 μL). Spin antibodies at maximal speed in a tabletop centrifuge for 2 min before use to avoid aggregates.

For 1 coverslip: 

3.2 μL mouse anti-IdU 

1.6 µL rat anti-CldU

35.2 µL Block Aid

57. Place coverslips on a staining rack. Wash for 5 min with blocking buffer.
58. Incubate with secondary antibodies for 50–60 min at RT:

For 1 coverslip: 

0.8 µL anti-mouse Cy3 (1:50)

0.8 µL anti-rat BV480 (1:50)

38.6 µL Block Aid

59. Place coverslips on staining rack and wash 5 min with blocking buffer.
60. Incubate with single-stranded DNA primary antibody for 1 h at RT:

For 1 coverslip:

2 µL mouse anti-ssDNA (1:40)

38 µL Block Aid

61. Place coverslips on staining rack and wash for 5 min with blocking buffer.
62. Incubate with secondary antibody for 45–60 min at RT:

For 1 coverslip:

0.8 µL anti-mouse AF-488 (1:50)

39.2 µL Block Aid

63. Place coverslips on staining rack and wash 15 min with blocking buffer, gentle shaking.
64. Wash coverslips in PBS for 5 min.
65. Dehydrate coverslips in 70%, 90% and 100% EtOH, 30–60 s each, air-dry.
66. Mount with 10 μL ProlongGold and cure overnight at RT in the dark.

#### 2.1.6. Imaging

The slides were scanned on Opera Phenix or Operetta CLS high-content imaging systems (Revvity, UK, formerly Perkin Elmer Ltd.). The Opera scans were used for analysis.

The images were acquired with the 40× N.A. 1.1 water immersion objective using the non-confocal scanning mode. Binning was set at 1, giving a resolution of 0.33 mm/pixel and a field size of 500 × 500 pixels.

The channels and filters used were:

Cyan (CldU): Opera: ex 425 nm, em 435–480 nm; Operetta: ex 435–460 nm, em 473–491 nm.

Alexa 488 (DNA): Opera: ex 488 nm, em 500–550 nm; Operetta: ex 460–490 nm, em 500–550 nm.

Cy3 (IdU): Opera: ex 561 nm, em 570–630 nm; Operetta: narrow band ex 530–560 nm, em 570–620 nm.

Alexa 647 (S9.6): Opera: ex 640 nm, em 650–760 nm; Operetta: ex 615–645 nm, em 655–760 nm.

Channels were imaged sequentially in Z-stacks with a step of 0.5 μm.

#### 2.1.7. Quantitation and Statistical Analysis

Image analysis was performed using Harmony 5.1 high content analysis software (Revvity, UK). Three planes were selected consisting of the sharpest focal plane and one either side, and a maximum projection image was created. Hybrid and DNA quantitation and analysis were performed using the following novel analysis protocol.

Note: The parts of the analysis pipeline highlighted in grey refer to an alternative way of hybrid spot detection that uses absolute intensity threshold to define hybrids. This could be useful in cases of e.g., higher staining background. Otherwise, these steps can be skipped. The quantitation in this study was performed using the Find Spots function.

Input ImageInput
Channel group: 1 Sequences: ALL Flatfield Correction: Basic Brightfield Correction Stack Processing: Maximum ProjectionFind Texture RegionsInputMethodOutput
Channel: Alexa 488 ROI: NoneMethod: Split into Classes Number of Classes: 2 Texture Scale: 1 px Region Scale: 1 px Training Region Radius: 2 px Include Intensity InformationOutput Population A: DNA Output Population B: Background (Figure S2)Calculate Morphology PropertiesInputMethodOutput
Population: DNA Region: RegionMethod: Standard AreaProperty Prefix: RegionCalculate Morphology Properties (2)InputMethodOutput
Population: Background Region: RegionMethod: Standard AreaProperty Prefix: RegionCalculate Intensity PropertiesInputMethodOutput
Channel: Alexa 488 Population: DNA Region: RegionMethod: Standard Mean Standard Deviation SumProperty Prefix: Intensity Region Alexa 488Calculate Intensity Properties (2)InputMethodOutput
Channel: Alexa 647 Population: DNA Region: RegionMethod: Standard Mean Standard Deviation SumProperty Prefix: Intensity Region Alexa 647Calculate Intensity Properties (3)InputMethodOutput
Channel: Alexa 647 Population: Background Region: RegionMethod: Standard Mean SumProperty Prefix: Intensity Region Alexa 647Filter ImageInputMethodOutput
Channel: Alexa 647Method: Sliding Parabola Curvature: 20Output Image: Sliding Parabola 647Find Image RegionInputMethodOutput
Channel: Sliding Parabola 647 ROI: DNA ROI Regin: RegionMethod: Absolute Threshold Lowest Intensity: ≥250 Highest Intensity: ≤inf Split into Objects Area: >5 px^2^Output population: Red/DNA Region Absolute threshold Output Region: Image RegionFind Image Region (2)InputMethodOutput
Channel: Sliding Parabola 647 ROI: Background ROI Regin: RegionMethod: Absolute Threshold Lowest Intensity: ≥250 Highest Intensity: ≤inf Split into Objects Area: >5 px^2^Output population: Red/Background Region Absolute threshold Output Region: Image RegionFind SpotsInputMethodOutput
Channel: Alexa 647 ROI: DNA ROI Region: RegionMethod: A Relative Spot Intensity: >0.07 Splitting Sensitivity: 1 Calculate Spot PropertiesOutput Population: Red spots in DNA regionFind Spots (2)InputMethodOutput
Channel: Alexa 647 ROI: Background ROI Region: RegionMethod: A Relative Spot Intensity: >0.07 Splitting Sensitivity: 1 Calculate Spot PropertiesOutput Population: Red spots in BackgroundDefine resultsResults


Method: List of Outputs Population: Red/DNA region Ab thresh Number of ObjectsPopulation: Red/Background Region Ab thresh Number of ObjectsPopulation: DNA Number of Objects Region Area [μm^2^]: Sum Intensity Region Alexa 488 Mean: Sum Intensity Region Alexa 488 Sum: Sum Intensity Region Alexa 647 Mean: Sum Intensity Region Alexa 647 Sum: Sum Number of Spots: Sum Number of Spots per Area of Region: SumPopulation: Red spots in DNA region Number of Objects Apply to All: NonePopulation: Background Region Area [μm^2^]: Sum Intensity Region Alexa 647 Mean: Sum Intensity Region Alexa 647 Sum: Sum Number of Spots: Sum Number of Spots per Area of Region: SumPopulation: Red spots in background Number of ObjectsMethod: Formula Output Formula: a/b Population Type: Objects Variable a: DNA—Intensity Region Alexa 488 Sum Sum Variable b: DNA—Region Area [μm^2^] Sum Output Name: DNA 488 sum/areaMethod: Formula Output Formula: a/b Population Type: Objects Variable a: Background—Intensity Region Alexa 647 Sum Sum Variable b: Background—Region Area [μm^2^] Sum Output Name: Background: Red sum/Background areaMethod: Formula Output Formula: a/b Population Type: Objects Variable a: DNA—Intensity Region Alexa 647 Sum Sum Variable b: DNA—Region Area [μm^2^] Sum Output Name: DNA: Red sum/DNA areaMethod: Formula Output Formula: a/b Population Type: Objects Variable a: DNA—Number of Spots Sum Variable b: DNA—Region Area [μm^2^] Sum Output Name: DNA: Red Spots/DNA areaMethod: Formula Output Formula: a/b Population Type: Objects Variable a: Red spots in background—Number of Objects Variable b: Background—Region Area [μm^2^] Sum Output Name: Background: Red Spots/Background areaMethod: Formula Output Formula: a/b Population Type: Objects Variable a: Red/DNA region Absolute threshold—Number of Objects Variable b: DNA—Region Area [μm^2^] Sum Output Name: Bright Red Spots/DNA areaMethod: Formula Output Formula: a/b Population Type: Objects Variable a: Red/Background Region Absolute threshold—Number of Objects Variable b: Background—Region Area [μm^2^] Sum Output Name: Bright Red Spots/Background areaObject Results Population: Red/DNA region Ab thresh: None Population: Red/Background Region Ab thresh: None Population: DNA: ALL Population: Red spots in DNA region: None Population: Background: None Population: Red spots in background: None

## 3. Results and Discussion

### 3.1. RNA:DNA Hybrids and Replication Forks Occur Independently of Each Other

**Figure 1:**
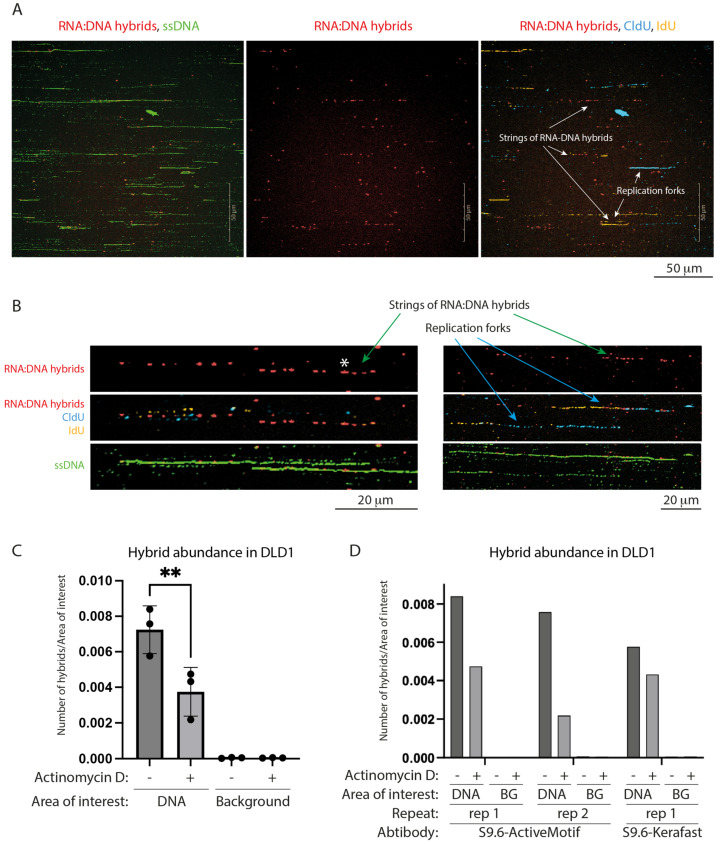
Simultaneous visualization of replication forks and RNA:DNA hybrids in a DNA combing assay. (**A**) Example fields of view of combed DNA, stained for single-stranded DNA (green), RNA:DNA hybrids/R-loops (red), and replication forks: CldU (cyan), followed by IdU (yellow). RNA:DNA hybrids and replication forks can appear independently or colocalize. (**B**) Zoomed-in examples of co-staining of RNA:DNA hybrids and replication forks in combed DNA. The dashed hybrid staining indicates that the structures are beyond the resolution limit. Asterisk: one of the larger intact hybrids observed with a length of about 3.5 kb. (**C**) Effect of transcription inhibition with actinomycin D on RNA:DNA hybrid abundance. 10 μM Actinomycin D was added to cells for 1 h prior to as well as during cell labelling. The number of hybrid spots normalized to DNA or Background area in a technical triplicate are plotted as Mean +/− SD. Statistical difference between non-treated (−) and actinomycin D-treated (+) cells is calculated by one-way ANOVA, (∗∗) adjusted *p*-value = 0.0054. Hybrids are identified exclusively in the DNA area and their abundance is reduced upon transcriptional inhibition. Background = non-DNA area. (**D**) Breakdown of technical triplicates in (**C**) by repeat and S9.6 antibody source.

**Figure 2:**
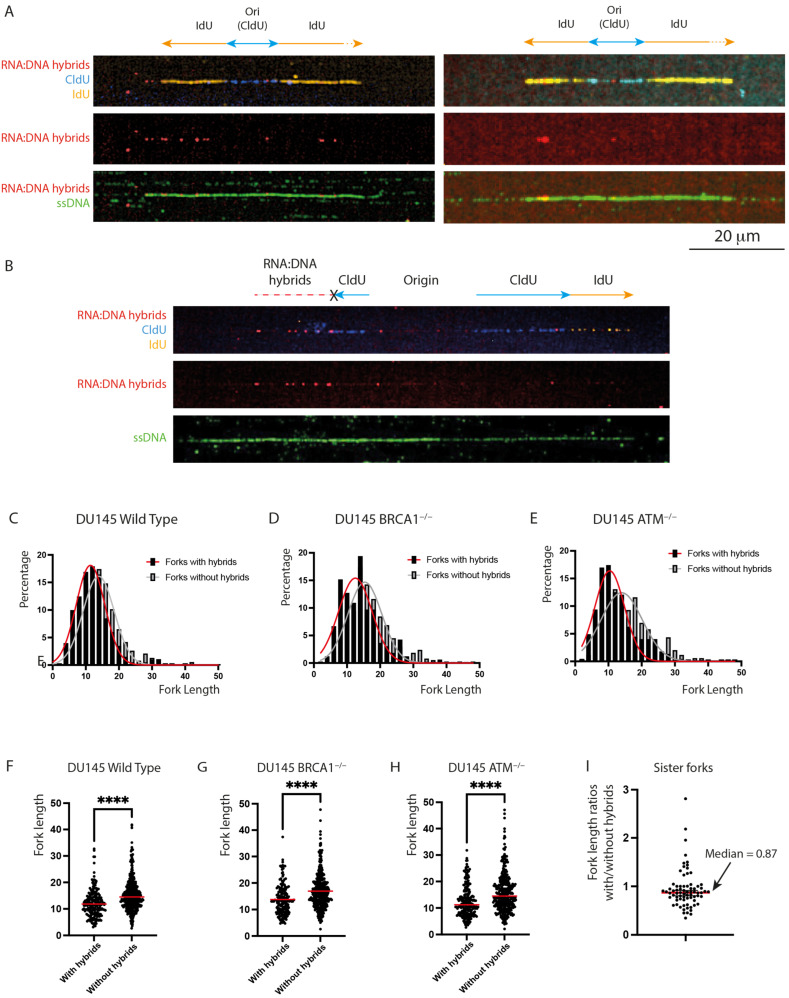
Direct measurement of the inhibitory effect of RNA:DNA hybrid/R-loop collisions on replication fork progression. (**A**) Two examples of sister forks of different lengths correlated with RNA:DNA hybrid/R-loop colocalization. Left: The left fork, which interferes with more hybrids, is shorter than its sister, which interferes with fewer hybrids. Right: The left fork, which interferes with hybrids is shorter than its sister, which is unchallenged by hybrids. The brightness and contrast of the entire image are adjusted in Photoshop. (**B**) An example of a replication fork collapse at a cluster of RNA:DNA hybrids/R-loops. The left sister fork collapses at a stretch of hybrids while the right sister fork progresses unimpeded. The brightness and contrast of the entire image are adjusted in Photoshop. (**C**) Frequency distribution of replication fork lengths (IdU) in DU145 Wild type grouped based on colocalization with at least one hybrid dot (black) or free of hybrids (grey). Manual measurement with FIJI. Gaussian fitting for visualization purposes. (**D**) Frequency distribution of replication fork lengths (IdU) in DU145 BRCA1 knockout grouped based on colocalization with at least one hybrid dot (black) or free of hybrids (grey). Manual measurement with FIJI. Gaussian fitting for visualization purposes. (**E**) Frequency distribution of replication fork lengths (IdU) in DU145 ATM knockout grouped based on colocalization with at least one hybrid dot (black) or free of hybrids (grey). Manual measurement with FIJI. Gaussian fitting for visualization purposes. (**F**) Violin plot visualisation of the data from (**C**), based on 586 manually measured forks (201 with hybrids, median = 11.87 AU; 385 without hybrids, median = 14.54 AU). Mann Whitney test—Significant, *p* value < 0.0001 (∗∗∗∗). (**G**) Violin plot visualisation of the data from (**D**), based on 544 manually measured forks (165 with hybrids, median = 13.07 AU; 379 without hybrids, median = 15.74 AU). Mann Whitney test—Significant, *p* value < 0.0001 (∗∗∗∗). (**H**) Violin plot visualisation of the data from (E), based on 569 manually measured forks (224 with hybrids, median = 11.21 AU; 345 without hybrids, median = 14.54 AU). Mann Whitney test—Significant, *p* value < 0.0001 (∗∗∗∗). (**I**) Ratios between lengths of sister forks, pooled from the three cell lines analysed in (**C**–**E**). Only pairs in which one fork colocalizes with hybrid(s) and its sister is free of hybrids are analysed. The median ratio between sisters with hybrids and without hybrids is 0.87. Based on 73 sister pairs. Forks with hybrids tend to be shorter than their sisters without hybrids within similar chromatin context.

The RNA:DNA hybrid signal and replication forks occur mostly independently of each other, indicating that the hybrids are not of Okazaki primer origin. In most cases, the replication forks are free of hybrids and, conversely, most hybrid staining occurs on DNA regions free from replication forks. Sometimes, the two signals overlap in agreement with co-occurrence and dynamic interactions between the transcription and replication machineries (Figure 1A). In fact, out of 1700 individual replication forks measured in this study (Figure 2), 102 have a hybrid dot at the tip and these include both forks which only overlap with one dot (65, or 3.8%) and forks which run through a cluster of hybrids. A fraction of the CldU/IdU tracks colocalized with multiple hybrids in agreement with single molecule studies showing that hybrids distinct from Okazaki fragments accumulate behind the fork [18].

All these indicate that the RNA:DNA hybrid staining that we observe is of transcriptional origin, most likely R-loops. Indeed, when transcription is inhibited with actinomycin D, which stops new transcription initiation, the number of hybrids decreases significantly (Figure 1C,D). In addition, if deproteinated agarose plugs are treated with RNAse H before combing, the abundance of hybrids also decreases (Figure S1A–D). Note that some R-loops are reported to be RNAse H-resistant [20,21].

In our combing assay, hybrids stain as dots and almost exclusively colocalize with DNA. Often, they appear in clusters and look like strings of dots. This likely reflects discrete transcription units (Figure 1A,B and Figure S1C). Short hybrid lines can also be observed (Figure 1B), indicating that their lengths are beyond the imaging resolution. The longest hybrid that we measured was ~5.8 kb (3.5 μm long; assuming replication rate of 1 kb/min). This agrees with reported variable R-loops’ lengths spanning between lower hundreds and a few thousand base pairs [20,22]. Most R-loops range between 100 and 500 bp—lengths, which would appear as dots with our imaging setting.

### 3.2. RNA:DNA Hybrid DNA Combing Protocol Complements Electron Microscopy Studies

R-loops and replication forks have been visualized in a single molecule level using electron microscopy (EM) [18]. Our combing assay overcomes the inherent EM restrictions of limited distance from the fork (about 15 kb) that is due to restriction digestion, as well as the limitation that small hybrids can escape detection.

### 3.3. Direct Visualization of Transcription-Replication Interference

This assay allows direct differentiation on a single molecule level of replication forks challenged with R-loops/transcription collisions (transcription-replication conflicts). Because the forks, which colocalize with hybrids, face physical and topological obstacles (they need to stall, remodel, and restart), the prediction is that they should on average be shorter. We observed events such as fork slowing due to hybrid collision (examples: Figure 2A) or even a fork collapse at a dense cluster of hybrids (example: Figure 2B).

To demonstrate the relationship between fork length and hybrid colocalization, we manually measured the fork lengths in DU145 WT/BRCA1 knockout/ATM knockout cell lines. Only ongoing replication forks were analyzed, i.e., IdU (second label) was measured if it was preceded with CldU signal and was found on an intact DNA fiber based on ssDNA stain. Additionally, details about the number of hybrid dots that colocalized with IdU as well as which forks were sisters (radiate out of the same origin), were recorded. Consistently, in all three cases we observed a clear fork length population differentiation based on hybrid colocalization. Forks that colocalize with hybrids are shorter than those which are free of hybrids (Figure 2C–E) and the differences were strongly significant (Mann-Whitney, *p* < 0.0001; Figure 2F–H). The number of hybrid dots per fork varied between 1 and 8, about 90% of which were up to 3.

Both replication fork speed and hybrid/R-loop formation depend on chromatin structure. As forks emanating from one origin tend to proceed with the same velocities [23], to take chromatin context out of consideration in the relationship between hybrid colocalization and fork speed, we focused specifically on those sister forks in which one of the sisters colocalized with hybrids and the other did not. Among all measured forks, we identified 73 such pairs and plotted the ratios of their lengths (Figure 2I). Consistent with the predictions and the population measurements, most ratios were below 1, with a median ratio of 0.87, i.e., the median challenged fork is 13% shorter than its sister fork. Few pairs had ratios larger than 1, indicating additional factors different from hybrids challenging those forks.

### 3.4. Sources of RNA:DNA Hybrids

RNA:DNA hybrids in the genome can originate from: (1) R-loops, (2) catalytic center of transcribing RNA polymerases, (3) DNA replication primers at replication origins and Okazaki fragments, and (4) long non-coding RNAs, such as TERRA. Most of these would in theory provide sufficiently long S9.6 binding sites of at least 6 base pairs [24].

Our data indicates that the hybrid signal that we observe results from active transcription, i.e., R-loops and potentially RNA polymerase elongation complexes. It is unknown if the short hybrids that form in the catalytic center of RNA polymerases persist after deproteination and genomic DNA preparation. Yet, potential colocalization between such hybrids and replication forks would represent transcription-replication conflicts, if not strictly R-loop collisions.

### 3.5. Quality of Staining

The quantitation of hybrid abundance strongly depends on the quality of both hybrid and DNA staining on combed slides since the amount of DNA is used to normalize the number of detected hybrids. The method has been successfully applied using S9.6 antibodies produced by two different companies with similar results (note that there are multiple suppliers of S9.6 antibodies). It is essential to test and validate antibody performance because various formulations and batches may perform differently. Similarly, single-stranded DNA staining needs to be consistent. This can be affected by the degree of denaturation and the quality of ssDNA antibody. However, interexperimental variability is less relevant when examining single molecule events within the same slide, such as in Figure 2C–I, since the presence of different populations of replication forks serve as internal controls.

In addition, imperfect ssDNA staining can make fibers appear as strings of dots. In such cases, hybrids can localize between these DNA dots and be excluded from the DNA area, even though they lie on the same line (fiber), leading to false negative hybrid-DNA colocalization (Figure S2C). Further development of automated and/or AI-based algorithms would allow operations such as the unidirectional extension of dotty DNA staining into an intact fiber and more precise colocalization analysis.

### 3.6. DNA Combing Using In-House Reagents

This protocol is largely based on using FiberPrep DNA Extraction Kit from Genomic Vision. However, the company and its reagents are, at the time of preparing this manuscript, no longer available. Importantly, alternatives exist, such as well-established DNA combing protocols using in-house reagents [25]. In addition, silanized combing coverslips are still commercially available.

## 4. Troubleshooting

### 4.1. Problem 1

No (or too little) DNA on the coverslips (related to steps 9–23)

Quality check should be performed after DNA combing. Potential reasons for lack of DNA on the slides can be:

- No or too few cells in the plug. In an extreme occasion when the cell number cannot be increased, such as in the case of precious and/or difficult to grow cells, the same coverslip could be re-combed in the same reservoir to accumulate more DNA fibres.
- Too many cells in the plug: this can lead to excessive entanglement and bundling of the genomic DNA after melting of the plug, which prevents it from diffusing in the volume of the reservoir.
- Insufficient incubation after pouring the DNA solution in the reservoir.

### 4.2. Problem 2

Too much DNA on the coverslips (related to step 9)

- Too many cells in the plug. When DNA fibres are too dense, this can lead to difficulties with the segmentation during analysis.

### 4.3. Problem 3

Focal planes differ at different areas of the coverslip (related to step 66)

- The coverslip is tilted during mounting. Extreme care must be taken to avoid dust particles when mounting the coverslips on the slides. Clean the slides carefully and use an air blower to remove any dust from the coverslips. If tilting still happens, perform the quantitation separately for areas of the coverslip with different focal planes.

### 4.4. Problem 4

No RNA:DNA hybrids signal (related to step 37)

- Test different manufacturers and batches of S9.6 antibodies to identify working lots before proceeding to fork staining. We have been successful with S9.6 antibodies from Kerafast (Cat#ENH002, Lot#202301) and ActiveMotif (Cat#65683, Lot#35021002). It should be noted that freeze-thaw cycles can be detrimental for S9.6 antibody performance in a combing assay. This can be overcome by preparing a large amount of working solution (1:500 in blocking buffer), which can be aliquoted and snap-frozen in one-time-use doses. From the antibodies used in this study, Kerafast S9.6 was very sensitive to freeze-thawing, in which case preserving it by aliquoting as described above was critical and sufficient to stabilise the antibody for repetitive usage; the ActiveMotif antibody was not noticeably sensitive to freeze-thawing, likely due to its formulation, which does not freeze at −20 °C.

### 4.5. Problem 5

Poor DNA staining (related to steps 52 and 60)

Efforts must be made to achieve good DNA staining, as it is used for normalization of the hybrid signal between slides. This can be optimized by:

- Testing different ssDNA antibodies.
- Increasing the incubation time and/or concentration of the ssDNA antibody.
- Ensuring sufficient denaturation of DNA. Instead of using NaOH, DNA can also be denatured with 2.5 M HCl for 25 min at room temperature. Either way, denaturation leads to a decrease in the S9.6 signal intensity. Care should be taken to denature enough, but not too extensively. In our experience, the S9.6 staining resists NaOH denaturation slightly better than HCl denaturation.

### 4.6. Problem 6

Technical variability in RNA:DNA hybrid abundance (related to Quantitation and Statistical Analysis)

Reliable quantitation of hybrids depends on both hybrid and DNA staining (Section 4.4 and Section 4.5)

- We recommend performing biological and technical repeats to strengthen the significance of any inter-sample differences in hybrid abundance.

## Figures and Tables

**Table 1:** Key resources table.

REAGENT	SOURCE	IDENTIFIER	COMMENT
**Antibodies**			
S9.6	Kerafast (USA)	ENH002	Lot 202301
S9.6	ActiveMotif (Belgium)	65683	Lot 35021002
Goat anti-mouse Alexa Fluor 647	Thermo Fisher Scientific (USA)	A-21236	
Mouse anti-BrdU antibody	BD Biosciences (USA)	347580	Stains IdU
Rat anti-BrdU antibody	Abcam (UK)	Ab6326	Stains CldU
Anti-mouse Cy3	Rockland (USA)	610-404-040	
Anti-rat BV480	BD Biosciences	564878	
Mouse anti-ssDNA	Developmental Studies Hybridoma Bank (USA)	Anti-ssDNA	Lot 5/11/17–37 ug/mL
Anti-mouse Alexa Fluor 488	Thermo Fisher Scientific	A-11001	
**Chemicals and other reagents**			
CldU	Sigma (USA)	C6891-100MG	Lot MKCJ7518
IdU	Sigma	I7125-25G	Lot BCBP3111V
BSA	Sigma	A9647	
BlockAid	Thermo Fisher Scientific	B10710	
Triton-X100	Sigma	9002-93-1	
Tween-20	Sigma	P1379	
RNAse A	Thermo Fisher Scientific	12091021	
RNAse H	New England Biolabs (USA)	M0297L	
4% Paraformaldehyde solution in PBS	Thermo Fisher Scientific	J19943.K2	
NaOH	Thermo Fisher Scientific	S318	
Ethyl alcohol	Thermo Fisher Scientific	BP28184	
ProlongGold	Thermo Fisher Scientific	P10144	

**Table 2:** Equipment.

EQUIPMENT	SOURCE	IDENTIFIER	COMMENT
Combing coverslips	Genomic Vision (France)	COV-002-RUO	
FiberComb machine	Genomic Vision	ADMCS	
FiberPrep DNA Extraction Kit	Genomic Vision	EXTR-001	
Coverslip rack	Sigma	Z688568	
Opera Phenix	Perkin Elmer (USA)	NA	
Oven	NA		60 °C

## Supplementary Materials

The following supporting information can be downloaded at: https://www.mdpi.com/article/10.3390/genes15091161/s1. Figure S1: RNAse H treatment in vitro reduces RNA:DNA hybrid abundance; Figure S2: Quantitation of RNA:DNA hybrid/R-loop abundance using Harmony 5.1.
