# Supplementary material for: Simultaneous Visualization of R-Loops/RNA:DNA Hybrids and Replication Forks in a DNA Combing Assay
Source: Genes (Basel). 2024 Sep 3;15(9):1161. doi: 10.3390/genes15091161 (PMC11430951; doi:10.3390/genes15091161)

FIGURE LEGENDS

Figure S1: RNAse H treatment in vitro reduces RNA:DNA hybrid abundance. A. Schematic of the RNAse treatments of agarose embedded DLD1 BRCA2 knockout cells. B. Manual quantitation performed in FiberStudio software (Genomic Vision) of relative hybrid abundance from non-treated (NT) or RNAse H-treated plugs. 10 fields of view located at different positions on the scanned slides were analysed by manual measurement or the lengths of DNA fibers and counting of the hybrid dots. The total number of hybrids were normalized to the sum of the lengths of the DNA fibers. NT: 215.8 mb DNA and 435 hybrids; RNAse H: 198.5 mb DNA and 122 hybrids. N=1. C. Field of view examples showing clusters of hybrids. D. Field of view example which is hybrid-free.

Supplementary Methods: Related to Figure S1. RNAse H treatment was performed in plugs on deproteinated DNA. Notably, we have been unsuccessful with RNAse H treatment on combed slides. It is plausible that fixation to the coverslip surface prevents efficient RNAse H access to the hybrids. The plugs were prepared up to step 16 in the protocol. The deproteinated and extensively washed plugs were incubated with 500 l PBS containing 0.1 mg/ml RNAse A and gently shaken at 37 C for 1 hour. The plugs were washed once with PBS, each plug was incubated with 400 l 1xRNAse H buffer +/- 20 units RNAse H (NEB #M0297) with gentle shaking (lowest setting) at 37 C overnight. This was followed by steps 17 to 47 (plug melting, combing, and S9.6 staining) skipping step 34 (RNAse A treatment). Coverslips were then stained with YOYO dye, dried, and imaged in FiberVision scanner. Analysis was performed manually for 10 different fields of view per slide by measuring the total DNA fiber lengths and counting the hybrids dots that colocalize with DNA.

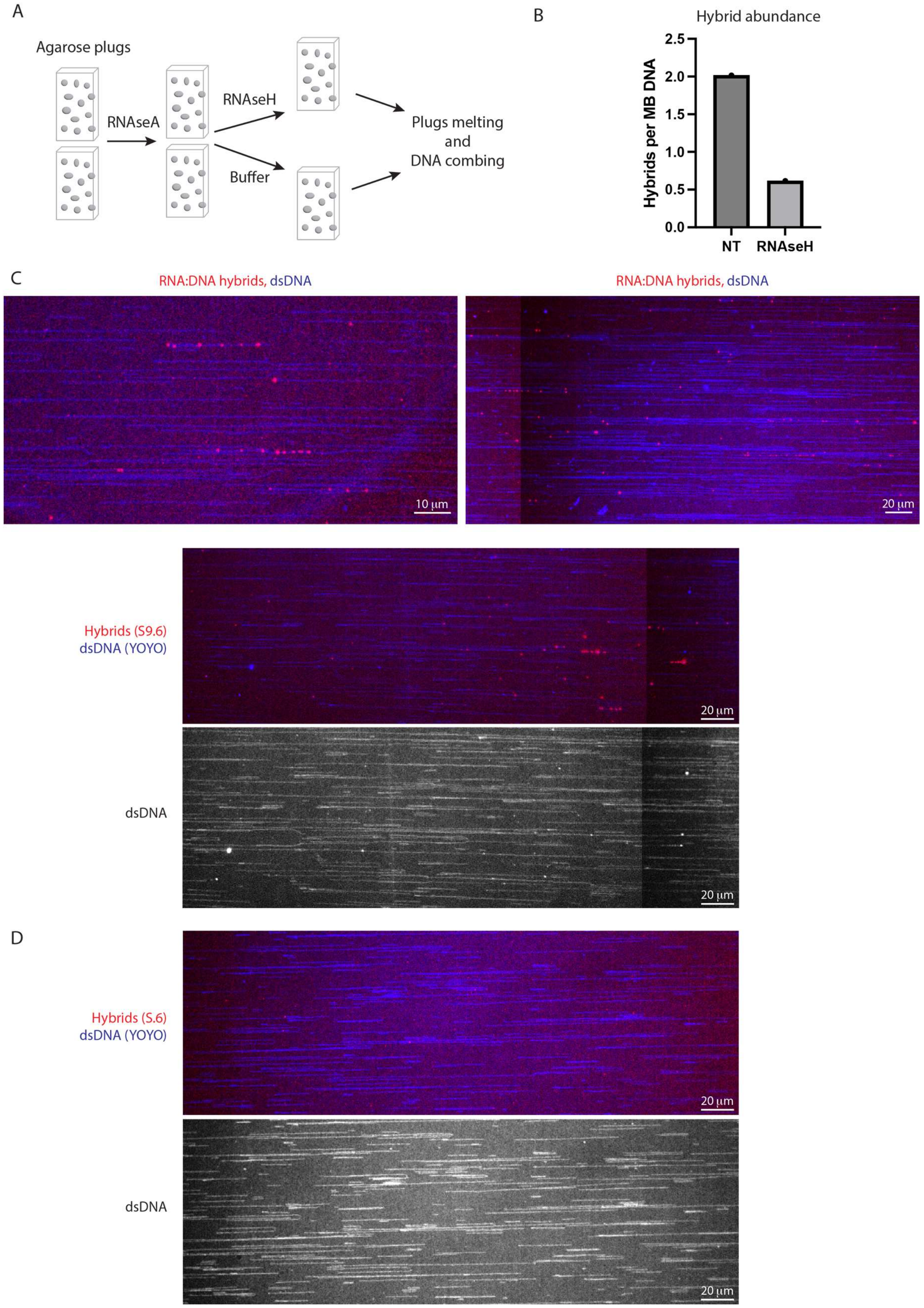
A
Agarose plugs
RNAseA
RNAseH
Buffer
Plugs melting
and
DNA combing
B
Hybrid abundance
Hybrids per MB DNA
2.5
2.0
1.5
1.0
0.5
0.0
NT
RNAseH
C
RNA:DNA hybrids, dsDNA
10 μm
RNA:DNA hybrids, dsDNA
20 μm
Hybrids (S9.6)
dsDNA (YOYO)
20 μm
dsDNA
20 μm
D
Hybrids (S.6)
dsDNA (YOYO)
20 μm
dsDNA
20 μm

Supplementary Figure S2: Quantitation of RNA:DNA hybrid/R-loop abundance using Harmony 5.1

A. Manual training of Harmony defines two regions on the slide: DNA and Background (i.e. non-DNA).

B. The total number of RNA:DNA hybrid dots (red spots) is normalized to the DNA area.

C. An example of an occasional 'false negative' colocalization between a hybrid and a DNA fibre, resulting from discontinuous DNA staining.

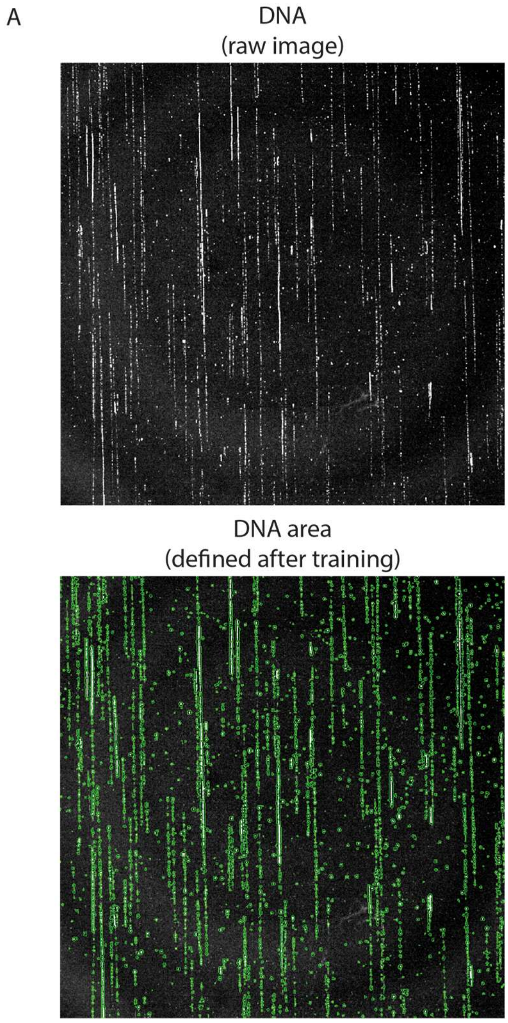

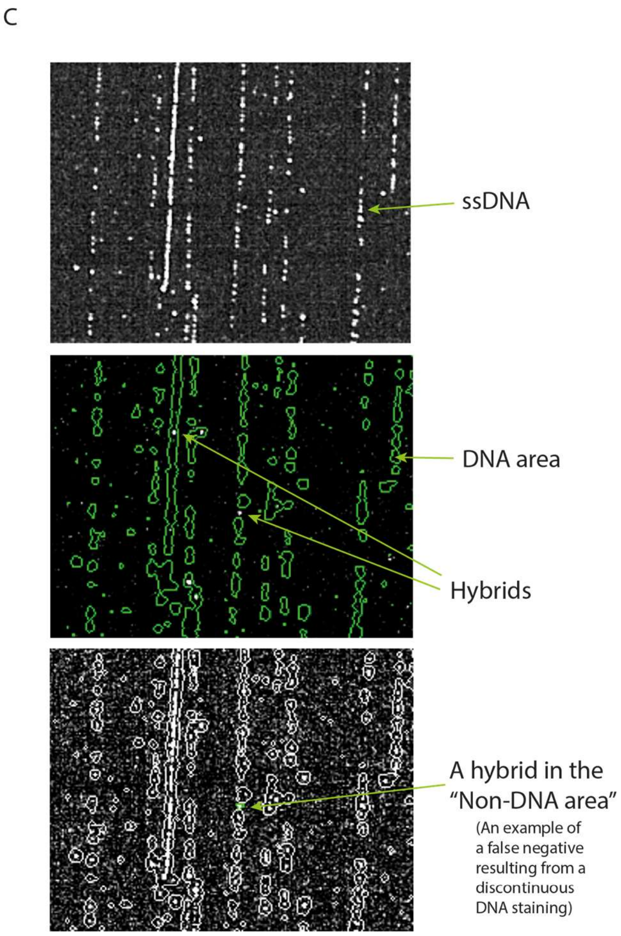

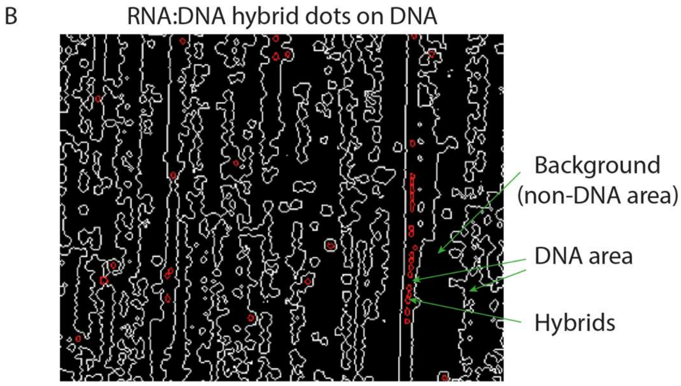

Supplement: Supplementary file 1 [file genes-15-01161-s001.zip › genes-3152318-supplementary.pdf]
